# Comparative Performance of the ANCLA Risk Score and Established Prognostic Models for Predicting ESKD in ANCA‐Associated Glomerulonephritis: A Latin American Cohort

**DOI:** 10.1155/ijne/5595479

**Published:** 2026-10-06

**Authors:** Joaquín Rodelo-Ceballos, Mauricio Restrepo-Escobar, Mayra Estacio, Carlos Muñoz-Vahos, Alejandra Taborda

**Affiliations:** ^1^ Division of Nephrology Hospital San Vicente Fundación Medellín Colombia; ^2^ Department of Internal Medicine Division of Nephrology University of Antioquia Medellín Colombia udea.edu.co; ^3^ Department of Internal Medicine Division of Rheumatology University of Antioquia Medellín Colombia udea.edu.co; ^4^ Division of Rheumatology Hospital San Vicente Fundación Medellín Colombia; ^5^ Department of Pathology University of Antioquia Medellín Colombia udea.edu.co

**Keywords:** ANCA-associated vasculitis, end-stage kidney disease, kidney biopsy, Latin American cohort, pauci-immune glomerulonephritis, prognostic score, risk stratification

## Abstract

**Background:**

Prognostic tools for kidney outcomes in ANCA‐associated vasculitis (AAV) were developed mainly in European and North American populations, and their performance in Hispanic populations remains less well characterized. ANCA in Latin America (ANCLA) was previously developed and internally validated in a Colombian cohort to predict end‐stage kidney disease (ESKD).

**Objective:**

To compare the prognostic performance of ANCLA with the Berden classification, Renal Risk Score (RRS), Mayo Clinic Chronicity Score (MCCS), and the ANCA Kidney Risk Score (AKRiS) for ESKD prediction in ANCA‐associated glomerulonephritis.

**Methods:**

This secondary comparative analysis used the previously described ANCLA derivation cohort of 164 adults with biopsy‐proven pauci‐immune necrotizing and/or crescentic glomerulonephritis treated from 2011 to 2024 at a tertiary referral center in Colombia. The five prognostic systems were evaluated in the same population using Kaplan–Meier analyses, Harrell’s C‐index, and 1‐ and 2‐year ROC analyses. Pairwise AUC differences between ANCLA and each comparator were formally assessed.

**Results:**

During a median follow‐up of 12.9 months, 63 patients (38.4%) progressed to ESKD. ANCLA showed the most favorable overall discrimination (C‐index 0.801; AUC 0.890 at 1 year and 0.896 at 2 years), followed by AKRiS (C‐index 0.760) and RRS (0.749); MCCS (0.673) and Berden (0.640) showed lower discrimination. ANCLA had significantly higher AUCs than MCCS and Berden at both horizons, whereas differences versus AKRiS and RRS were smaller. ESKD occurred in 7.3% of the ANCLA low‐risk group and 82.5% of the very‐high‐risk group.

**Conclusion:**

In this high‐risk, predominantly Hispanic Latin American cohort, ANCLA showed the most favorable short‐term discrimination, while AKRiS and RRS also performed well. Independent external validation remains necessary.

**Summary at a glance**
• Prognostic models for kidney outcomes in ANCA‐associated vasculitis have limited validation in Latin American populations. In a head‐to‐head analysis within the cohort in which ANCLA was originally developed, ANCLA showed the highest numerical short‐term discrimination, although performance was closer to AKRiS and RRS than to MCCS or the Berden classification. These findings provide comparative benchmarking of available prognostic systems and support independent external validation of ANCLA.

## 1. Introduction

Antineutrophil cytoplasmic antibody (ANCA)–associated vasculitis (AAV) is a systemic small‐vessel vasculitis in which kidney involvement is common and contributes substantially to morbidity and the risk of kidney failure [1]. Despite advances in immunosuppressive therapy, kidney involvement remains a major driver of long‐term morbidity, and early risk stratification is essential to guide treatment intensity, supportive care, and follow‐up planning. Kidney biopsy remains the cornerstone of prognostication, as histopathologic lesions provide critical information for predicting outcomes and guiding therapeutic decisions [2].

Over the past decade, multiple histopathologic classifications and prognostic models have been developed to estimate kidney outcomes in AAV. The Berden classification stratifies patients into four categories—focal, crescentic, mixed, and sclerotic—based solely on the distribution of glomerular lesions [2]. While this framework standardized histopathologic reporting, subsequent studies have reported limited prognostic discrimination, particularly due to overlap between crescentic and mixed classes [3]. Subsequent studies highlighted the need to assess prognostically relevant lesions beyond the predominant glomerular pattern [4]. Chronicity grading systems applied to AAV cohorts, including the Mayo Clinic Chronicity Score (MCCS), quantify irreversible injury across glomerular, tubulointerstitial, and vascular compartments [5]. The Renal Risk Score (RRS) integrates the percentage of normal glomeruli and interstitial fibrosis/tubular atrophy (IFTA) with baseline eGFR to stratify patients into low‐, intermediate‐, and high‐risk groups [6].

The ANCA Kidney Risk Score (AKRiS) was recently developed and validated in a multinational cohort of more than 1400 patients. AKRiS uses serum creatinine, normal glomeruli, and IFTA and includes a fourth, very‐high‐risk category [7]. In a large European cohort, Stella et al. found stronger predictive performance for RRS and MCCS than for the Berden classification [8], supporting the value of integrating clinical and histopathologic information.

A major gap, however, lies in the underrepresentation of Latin American populations in vasculitis research. Regional differences in genetic background, disease phenotype, environmental exposures, and healthcare access may affect the external validity of models derived primarily from European and North American cohorts. ANCA in Latin America (ANCLA) was previously developed and internally validated in a predominantly Hispanic Latin American cohort using age, baseline eGFR, 24‐hour proteinuria, and the percentage of normal glomeruli [9]. The original study focused on model development and internal validation, with exploratory comparisons limited to the Berden classification and RRS [9]. More recently, a comparative study from Mexico demonstrated clinically relevant differences in the performance of available prognostic scores, further supporting their evaluation across Latin American populations [10].

The aim of the present study was to evaluate and compare the prognostic performance of ANCLA, the Berden classification, RRS, MCCS, and AKRiS for predicting early (1‐2‐year) progression to end‐stage kidney disease (ESKD) in Latin American adults with biopsy‐proven ANCA‐associated glomerulonephritis.

## 2. Methods

### 2.1. Study Design and Population

We performed a secondary comparative prognostic analysis of the previously described ANCLA retrospective cohort at Hospital Universitario San Vicente Fundación, a tertiary referral center in Medellín, Colombia. Adults aged ≥ 18 years diagnosed between January 2011 and December 2024 were eligible.

The cohort was the same population used to derive and internally validate ANCLA [9]; no additional participants were enrolled. We included biopsy‐proven pauci‐immune necrotizing and/or crescentic glomerulonephritis within the clinicopathologic spectrum of AAV, including ANCA‐negative pauci‐immune disease considered clinically consistent with AAV. Diagnosis was established by a rheumatologist using clinical findings, ANCA serology, and kidney biopsy. Additional cohort details are reported elsewhere [9].

Exclusion criteria were biopsies with < 10 glomeruli, ESKD at presentation, concomitant systemic autoimmune disease, alternative diagnoses mimicking vasculitis, or insufficient data to compute the prognostic models. Patients were followed for at least 12 months unless ESKD occurred earlier [9].

### 2.2. Data Collection

Clinical and treatment data were obtained retrospectively from electronic records through December 2024. Baseline variables were defined within 30 days of kidney biopsy and included age, sex, self‐reported race and ethnicity, comorbidities, ANCA specificity, serum creatinine, eGFR calculated with the 2021 CKD‐EPI creatinine equation [11], hemoglobin, and proteinuria. Quantitative proteinuria was preferentially obtained from a 24‐hour collection; when unavailable, spot urine protein‐to‐creatinine ratio was used. BVAS was recorded when available [12].

Treatment variables included glucocorticoids, cyclophosphamide, rituximab, azathioprine, mycophenolate mofetil, methotrexate, and plasma exchange.

### 2.3. Histopathologic Assessment

Kidney biopsies were assessed by light microscopy with routine stains and immunofluorescence in all cases; electron microscopy was used selectively. A single expert nephropathologist, blinded to clinical outcomes, performed the histopathologic assessment as previously described [9]. Glomerular lesions followed Renal Pathology Society consensus definitions [13], and systematic assessment of glomerular and tubulointerstitial injury was supported by prior AAV prognostic studies [14].

Variables required by the evaluated models included percentage of normal glomeruli, glomerulosclerosis, crescents, IFTA, and vascular chronicity lesions. Normal glomeruli had no sclerosis, crescents, or fibrinoid necrosis. IFTA was graded semiquantitatively by cortical area using standardized categories [15]; its prognostic relevance in AAV is well established [16]. Additional morphologic details are reported in the ANCLA derivation study [9].

To ensure comparability across models, histopathologic variables were extracted in a standardized manner, and each prognostic system was applied according to the definitions and scoring rules of its original publication, consistent with prior comparative studies [8, 10].

### 2.4. Outcome Definition

The primary outcome was ESKD, defined as initiation of maintenance dialysis or sustained eGFR < 15 mL/min/1.73 m^2^ for ≥ 3 months [9]. Time‐to‐event was measured from kidney biopsy to ESKD, last follow‐up, death, or study end.

### 2.5. Prognostic Models

Each patient was classified according to five prognostic systems, implemented strictly as originally described by their developers, using baseline clinical and histopathologic variables obtained near the time of kidney biopsy. Patients were categorized according to the Berden histopathologic classification into focal, crescentic, mixed, or sclerotic classes based on the distribution of glomerular lesions [2]. Chronic damage was additionally quantified using the MCCS, applying the published definitions used in AAV cohorts, which integrate glomerulosclerosis, IFTA, and vascular chronic injury [5]. The RRS was calculated by combining baseline kidney function (eGFR at biopsy) with histopathologic parameters (percentage of normal glomeruli and IFTA), and patients were stratified into low‐, intermediate‐, and high‐risk categories according to the original scoring system [6]. The AKRiS was computed using serum creatinine, percentage of normal glomeruli, and IFTA and categorized into low, moderate, high, and very high‐risk groups following the authors’ published thresholds [7]. Finally, the ANCLA score was calculated using the original formula derived from four continuous predictors (age, baseline eGFR, 24‐hour proteinuria, and percentage of normal glomeruli) [9]. ANCLA was analyzed continuously for discrimination. For categorical analyses and Kaplan–Meier estimates, the previously established ANCLA risk categories were used: low (< 4), moderate (≥ 4 to < 15), high (≥ 15 to < 29), and very high (≥ 29) [9].

### 2.6. Statistical Analysis

Baseline characteristics were summarized as medians (interquartile range [IQR]) for continuous variables and as counts (percentages) for categorical variables. Between‐group comparisons (ESKD vs. no ESKD) were performed using the Mann–Whitney *U* test for continuous variables and *χ* ^2^ or Fisher’s exact test for categorical variables, as appropriate.

Comparative performance was evaluated using time‐to‐event methods consistent with prior AAV score comparisons [8–10]. Kidney survival across model‐defined categories was assessed with Kaplan–Meier curves and log‐rank tests. Discrimination was summarized by Harrell’s C‐index from Cox models; proportional hazards assumptions were assessed with Schoenfeld residuals. Horizon‐specific receiver operating characteristic (ROC) analyses were prespecified at 1 and 2 years; patients censored before a given horizon were excluded from that horizon‐specific ROC analysis.

Pairwise differences in horizon‐specific AUC between ANCLA and each comparator were formally assessed using DeLong’s test. Each pairwise comparison was conducted in a common paired sample comprising only patients with sufficient follow‐up information at the corresponding horizon and complete data to calculate both ANCLA and the comparator score. Consequently, the AUCs used to calculate the paired differences may differ slightly from the model‐specific AUCs reported for the overall available samples. Ninety‐five percent confidence intervals for AUC differences were additionally estimated using paired bootstrap resampling. Multiplicity across the four ANCLA‐versus‐comparator comparisons at each horizon was controlled using the Holm procedure.

Calibration was assessed for ANCLA at common horizons of 1 and 2 years. Because ANCLA is supported by a prognostic equation and permits estimation of individual absolute risk, predicted probabilities were obtained from the survival framework using the estimated baseline survival function. Calibration‐in‐the‐large (intercept) and calibration slope were estimated at each horizon using inverse probability of censoring weighting (IPCW) and a complementary log‐log calibration model, consistent with the ANCLA derivation framework [9]. Formal calibration was not evaluated for the comparator models, which primarily define risk strata and do not provide individual absolute‐risk equations applicable at these horizons.

Bootstrap resampling (250 repetitions with replacement) was used to obtain optimism‐corrected performance estimates for ANCLA, consistent with the original derivation framework [9]. Analyses were conducted on a complete‐case basis for variables required to compute each score; patients with missing components for a given prognostic system were excluded only from that model‐specific analysis.

Statistical analyses were performed using Stata v17.0 (StataCorp, College Station, TX, USA) and R 4.5.2 (R Foundation for Statistical Computing, Vienna, Austria). A two‐sided *p* value < 0.05 was considered statistically significant.

## 3. Results

### 3.1. Patient Characteristics and ESKD Outcomes

A total of 164 adults with biopsy‐proven pauci‐immune necrotizing and/or crescentic glomerulonephritis consistent with AAV were included. Median age was 59.5 years (IQR 42.5–68), and 47.0% were female. Most participants self‐identified as Hispanic (88%), and 12% reported Afrodescendant ancestry. At diagnosis, the cohort presented with advanced kidney involvement, with a median baseline eGFR of 15 mL/min/1.73 m^2^ (IQR 8–34) and median 24‐hour proteinuria of 1.7 g/day (IQR 0.9–3.3). MPO‐ANCA was the most frequent serologic subtype (60.0%), followed by PR3‐ANCA (23.2%), while ANCA‐negative pauci‐immune glomerulonephritis accounted for 17.1% of cases. Median follow‐up was 12.9 months (IQR 0.4–41.5), reflecting a high burden of early adverse kidney outcomes in this referral population.

During follow‐up, 63 patients (38.4%) progressed to ESKD. Compared with patients who remained dialysis‐independent, those who developed ESKD were younger (median 56 vs. 61 years, *p* = 0.020), had substantially lower baseline eGFR (8 vs. 23 mL/min/1.73 m^2^, *p* < 0.001), and presented with higher proteinuria (2.6 vs. 1.5 g/24 h, *p* < 0.001) and lower hemoglobin levels (8.4 vs. 9.9 g/dL, *p* < 0.001). ANCA serotype distribution did not differ significantly between outcome groups. Extrarenal manifestations and systemic inflammatory measures, including BVAS, showed no relevant differences across ESKD strata.

Most patients received induction glucocorticoids, and cyclophosphamide was the most frequently used immunosuppressive agent. Cyclophosphamide use was significantly lower among patients who progressed to ESKD (57% vs. 81%, *p* = 0.001), whereas use of rituximab and plasma exchange was similar between groups. Kidney biopsies contained a median of 18 glomeruli (IQR 12–24). Progression to ESKD was strongly associated with chronic histopathologic injury, including a lower percentage of normal glomeruli (7% vs. 28%; *p* < 0.001), higher global glomerulosclerosis (44% vs. 13%; *p* < 0.001), and greater IFTA (38% vs. 20%; *p* < 0.001). Baseline clinical and histopathologic characteristics stratified by ESKD outcome are summarized in Table 1, while characteristics stratified by the composite outcome of ESKD or death are presented in Supporting Table S1.

**TABLE 1:** Baseline clinical and histopathologic characteristics according to progression to ESKD.

Variable	Total (*n* = 164)	No ESKD (*n* = 101)	ESKD (*n* = 63)	*p* value
Age, years, median (IQR)	59.5 (42.5–68)	61 (51–69)	56 (36–67)	0.020
Female sex, *n* (%)	77 (47.0)	44 (43.6)	33 (52.4)	0.271
BVAS, median (IQR)	17 (14–20)	17 (14–20)	17 (13–21)	0.768
eGFR (CKD‐EPI 2021), mL/min/1.73 m^2^, median (IQR)	15 (8–34)	23 (14–42)	8 (6–14)	< 0.001
Active urinary sediment, *n* (%)	155 (94.5)	95 (94.1)	60 (95.2)	0.747
24‐hour proteinuria, mg/24 h, median (IQR)	1749 (900–3265)	1490 (799–2364)	2565 (1700–4000)	< 0.001
ANCA subtype, *n* (%)				0.259
MPO	98 (60.0)	65 (64.4)	33 (52.4)	
PR3	38 (23.2)	22 (21.8)	16 (25.4)	
ANCA‐negative	28 (17.1)	14 (13.9)	14 (22.2)	
Hemoglobin, g/dL, median (IQR)	9 (7.9–10.4)	9.9 (8.1–10.8)	8.4 (7.1–9.2)	< 0.001
IV glucocorticoid pulses, *n* (%)	156 (95.1)	99 (98.0)	57 (90.5)	0.055
Cyclophosphamide use, *n* (%)	118 (72.0)	82 (81.2)	36 (57.1)	0.001
Rituximab use, *n* (%)	21 (12.8)	15 (14.9)	6 (9.5)	0.321
Plasma exchange, *n* (%)	40 (24.4)	24 (23.8)	16 (25.4)	0.813
Hypertension, *n* (%)	84 (51.2)	53 (52.5)	31 (49.2)	0.684
Diabetes mellitus, *n* (%)	26 (16.0)	14 (14.0)	12 (19.1)	0.391
Dyslipidemia, *n* (%)	24 (14.7)	15 (15.0)	9 (14.3)	0.900
Heart failure, *n* (%)	16 (9.8)	12 (12.0)	4 (6.4)	0.238
Cancer, *n* (%)	4 (2.5)	3 (3.0)	1 (1.6)	0.570
Total glomeruli, median (IQR)	18 (12–24)	18 (12–24)	18 (12–24)	0.778
Fibrous crescents, median (IQR)	0 (0–1)	0 (0–1)	0 (0–1)	0.894
IFTA (global), %, median (IQR)	20 (10–45)	20 (10–30)	38 (15–60)	< 0.001
Cellular crescents, median (IQR)	2 (0–5)	2 (1–5)	2 (0–5)	0.319
Fibrocellular crescents, median (IQR)	0 (0–1)	0 (0–1)	0 (0–2)	0.277
Fibrinoid necrosis, median (IQR)	1 (0–2)	1 (0–2)	0 (0–2)	0.042
Globally sclerotic glomeruli, median (IQR)	3 (1–9)	2 (0–6)	6 (2–11)	< 0.001
Glomerulosclerosis, %, median (IQR)	22 (5–54)	13 (0–35)	44 (20–69)	< 0.001
Normal glomeruli, %, median (IQR)	19 (2–40)	28 (11–50)	7 (0–17)	< 0.001
Arteritis, *n* (%)	18 (11.2)	16 (16.0)	2 (3.3)	0.013
Atherosclerosis, *n* (%)	75 (46.6)	39 (39.0)	36 (59.0)	0.014
Berden classification, *n* (%)				0.002
Focal	30 (18.3)	25 (24.8)	5 (7.9)	
Mixed	25 (15.2)	16 (15.8)	9 (14.3)	
Crescentic	66 (40.2)	43 (42.6)	23 (36.5)	
Sclerotic	43 (26.2)	17 (16.8)	26 (41.3)	
Renal Risk Score (RRS), *n* (%)				< 0.001
Low risk	27 (17.0)	27 (27.0)	0 (0)	
Intermediate risk	67 (42.1)	46 (46.0)	21 (35.6)	
High risk	65 (40.9)	27 (27.0)	38 (64.4)	
Mayo Clinic Chronicity Score (MCCS), *n* (%)				0.002
Minimal	31 (19.5)	27 (27.0)	4 (6.8)	
Mild	39 (24.5)	26 (26.0)	13 (22.0)	
Moderate	51 (32.1)	31 (31.0)	20 (33.9)	
Severe	38 (23.9)	16 (16.0)	22 (37.3)	
ANCLA score, *n* (%)				< 0.001
Low risk	41 (25.2)	38 (38.0)	3 (4.8)	
Moderate risk	41 (25.2)	31 (31.0)	10 (15.9)	
High risk	41 (25.2)	24 (24.0)	17 (27.0)	
Very high risk	40 (24.5)	7 (7.0)	33 (52.4)	
AKRiS, *n* (%)				< 0.001
Low risk	69 (42.3)	55 (54.5)	14 (22.6)	
Moderate risk	45 (27.6)	29 (28.7)	16 (25.8)	
High risk	25 (15.3)	15 (14.9)	10 (16.1)	
Very high risk	24 (14.7)	2 (2.0)	22 (35.5)	

*Note:* Baseline characteristics of the cohort stratified by progression to ESKD. Data are presented as median (interquartile range) for continuous variables and *n* (%) for categorical variables. *p* values correspond to Mann–Whitney *U* tests for continuous variables and *χ* ^2^ or Fisher’s exact tests for categorical variables, as appropriate. eGFR was calculated using the CKD‐EPI 2021 equation. Proteinuria is expressed as mg/24 h. ANCA, antineutrophil cytoplasmic antibody; MPO, myeloperoxidase; PR3, proteinase 3.

Abbreviations: AAV, ANCA‐associated vasculitis; BVAS, Birmingham Vasculitis Activity Score; eGFR, estimated glomerular filtration rate; ESKD, end‐stage kidney disease; IFTA, interstitial fibrosis/tubular atrophy.

### 3.2. Risk Stratification by Prognostic Systems

When outcomes were stratified according to each prognostic model (Table 2), all systems demonstrated clinically meaningful gradients in ESKD risk. Within the Berden histopathologic classification, ESKD occurred most frequently in the sclerotic class (60.5%) and least frequently in the focal class (16.7%). For the RRS, ESKD ranged from 0% in the low‐risk category to 58.5% in the high‐risk category. The MCCS similarly showed a stepwise increase in risk, from 12.9% in the minimal chronicity group to 57.9% in the severe chronicity group. The AKRiS provided broader stratification, with ESKD occurring in 20.3% of low‐risk patients and 91.7% of those classified as very high risk. Observed frequencies of the composite outcome (ESKD or death) across prognostic models are summarized in Supporting Table S2.

**TABLE 2:** Frequency of ESKD across prognostic model categories.

Prognostic model	Risk category	ESKD (%)
Berden classification	Focal	16.7
Berden classification	Mixed	36.0
Berden classification	Crescentic	34.8
Berden classification	Sclerotic	60.5
Renal Risk Score (RRS)	Low risk	0.0
Renal Risk Score (RRS)	Intermediate risk	31.3
Renal Risk Score (RRS)	High risk	58.5
Mayo Clinic Chronicity Score (MCCS)	Minimal	12.9
Mayo Clinic Chronicity Score (MCCS)	Mild	33.3
Mayo Clinic Chronicity Score (MCCS)	Moderate	39.2
Mayo Clinic Chronicity Score (MCCS)	Severe	57.9
ANCLA score	Low risk	7.3
ANCLA score	Moderate risk	24.4
ANCLA score	High risk	41.5
ANCLA score	Very high risk	82.5
AKRiS	Low risk	20.3
AKRiS	Moderate risk	35.6
AKRiS	High risk	40.0
AKRiS	Very high risk	91.7

*Note:* Observed frequency of ESKD within each risk category for the Berden classification, RRS, MCCS, AKRiS, and ANCLA. Percentages represent the proportion of individuals within each category who progressed to ESKD during follow‐up.

The ANCLA score demonstrated a particularly marked risk gradient, with ESKD occurring in 7.3% of patients in the low‐risk group and 82.5% in the very high‐risk group, demonstrating substantial separation across the predefined risk categories. Kaplan–Meier estimates of ESKD‐free survival across risk categories for all prognostic systems are shown in Figure 1.

**FIGURE 1:**
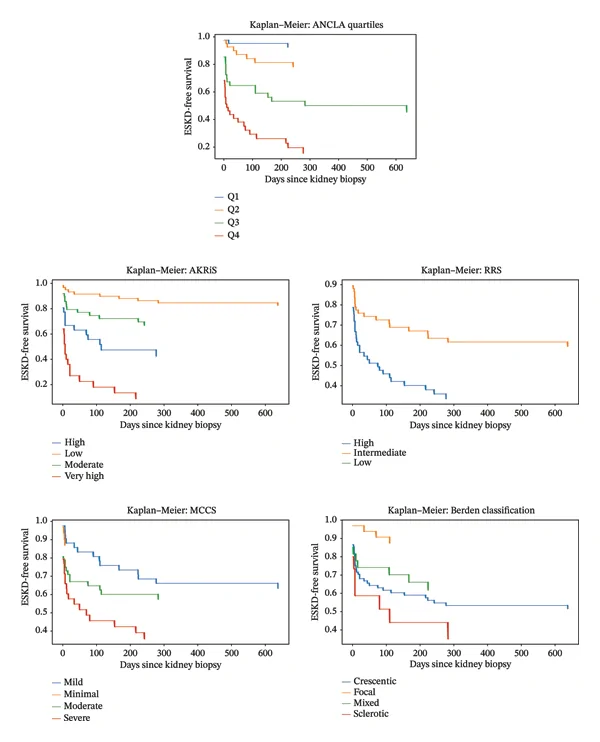
Kaplan–Meier curves for ESKD‐free survival across prognostic model categories. Kaplan–Meier estimates of ESKD‐free survival stratified by risk categories for ANCLA, AKRiS, RRS, MCCS, and the Berden classification. Time zero corresponds to the kidney biopsy date. Differences across strata were assessed using the log‐rank test.

### 3.3. Discrimination, Calibration, and Comparative Performance at 1‐2 Years

Across time‐to‐event analyses, ANCLA showed the most favorable overall discrimination (Harrell’s C‐index 0.801), followed by AKRiS (0.760) and RRS (0.749), whereas MCCS (0.673) and the Berden classification (0.640) showed lower discrimination. Kaplan–Meier curves confirmed significant separation of ESKD‐free survival across risk strata for all five models (log‐rank *p* < 0.001) (Figure 1).

Horizon‐specific ROC analyses at prespecified 1‐ and 2‐year horizons yielded consistent results. ANCLA had AUCs of 0.890 and 0.896 at 1 and 2 years, respectively. Corresponding AUCs were 0.841 and 0.843 for AKRiS, 0.828 and 0.839 for RRS, 0.740 and 0.733 for MCCS, and 0.698 and 0.693 for the Berden classification. Horizon‐specific ROC curves at 1 and 2 years are displayed in Figure 2.

**FIGURE 2:**
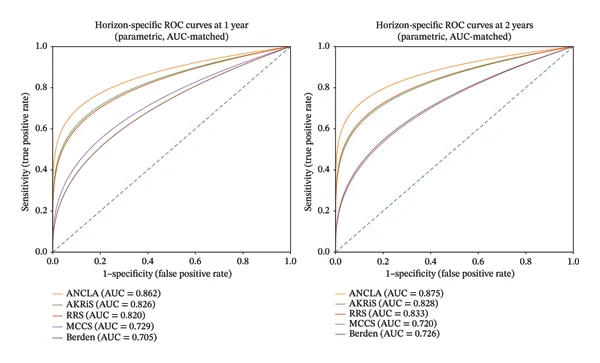
Horizon‐specific ROC curves at 1 and 2 years. Receiver operating characteristic (ROC) curves comparing discrimination at the 1‐ and 2‐year horizon for ANCLA, AKRiS, RRS, MCCS, and the Berden classification. ESKD was defined as occurring on or before 1 and 2 years after biopsy; individuals censored before the corresponding horizon were excluded from each horizon‐specific ROC estimation. AUC values are reported in the figure.

ANCLA showed the most favorable AUC estimates at both horizons. Pairwise comparisons demonstrated significantly greater discrimination versus MCCS and the Berden classification at 1 and 2 years. Differences versus AKRiS and RRS were smaller. At 1 year, the comparison with AKRiS was not statistically significant after Holm adjustment, whereas the difference versus RRS remained significant (adjusted *p* = 0.039). At 2 years, comparisons with both AKRiS and RRS were borderline after Holm adjustment (adjusted *p* = 0.050 for each). These findings indicate greater overlap in discrimination between ANCLA and the two strongest comparator models (Supporting Table S3). Detailed discrimination metrics for all models and calibration intercepts and slopes for ANCLA are presented in Supporting Table S4. A detailed description of model inputs, scoring rules, and risk category definitions is provided in Supporting Table S5.

## 4. Discussion

In this comparative evaluation of prognostic models for ANCA‐associated glomerulonephritis, ANCLA showed the most favorable short‐term discrimination for ESKD, with the clearest differences versus MCCS and the Berden classification. Performance was more closely aligned with AKRiS and RRS, indicating substantial overlap with the strongest established models.

The Berden classification remains a practical morphology‐based framework [2], but its discrimination is limited by overlap between intermediate classes and categorical representation of preserved nephron mass [3]. In our cohort, the sclerotic class retained prognostic value, although overall discrimination was lower than that with models incorporating clinical or chronicity information, consistent with other comparative cohorts [8, 10].

RRS and MCCS extend prognostication by incorporating kidney function and/or chronic damage across kidney compartments [5, 6, 17]. RRS showed strong discrimination, whereas MCCS was more modest despite a clear risk gradient. This may reflect greater short‐term prognostic resolution from combining baseline function with preserved glomeruli and IFTA in RRS [6], whereas MCCS primarily quantifies chronic injury [5].

AKRiS uses serum creatinine and reweighted histologic variables and has shown strong performance in multinational validation [7]. In our cohort, AKRiS performed strongly, with discrimination close to RRS and higher than MCCS and Berden. Recent comparative studies from Mexico and Portugal reported favorable discrimination for AKRiS, with RRS and MCCS also demonstrating prognostic value [10, 18]. Although ANCLA yielded higher discrimination estimates in our dataset, these differences were smaller when compared with AKRiS and RRS and should be interpreted in context. Our population had advanced kidney dysfunction at baseline, a high frequency of early ESKD events, and relatively short follow‐up, which may favor models optimized for near‐term risk. In contrast, AKRiS retains important strengths, including broad external validation and evidence of stable performance in diverse populations across longer horizons [7].

ANCLA may benefit from incorporating proteinuria and continuous predictors. Proteinuria has been associated with adverse kidney outcomes and reduced preserved nephron mass [19], while the percentage of normal glomeruli is a consistently informative marker of kidney reserve [20]. Independent European studies confirmed prognostic gradients according to preserved normal glomeruli [21] and better 1‐year kidney function with a higher proportion of normal glomeruli in severe AAV‐associated acute kidney injury [22]. Korean and Russian cohorts also linked chronic tubulointerstitial injury, particularly interstitial fibrosis and tubular atrophy, with adverse kidney outcomes [23, 24].

The inverse association between age and ESKD in our cohort should be interpreted cautiously. Age effects in AAV are influenced by competing risks, frailty, and mortality; in older adults, these factors substantially modify trajectories for ESKD, death, and severe infection [25]. Our findings may therefore reflect the high frequency of early kidney failure rather than a simple protective effect of older age.

Overall, our findings support multidimensional prognostic assessment integrating clinical, functional, and histopathologic information. Comparative European and Latin American studies similarly favor composite models over purely morphologic classifications [8, 10, 18]. Kidney prognosis in AAV reflects baseline function, histopathologic damage, and subsequent disease course [26], while regional tools such as the Japanese PACS further illustrate the value of combining clinical and histologic markers in specific populations [27].

Clinically, ANCLA may complement established tools by providing early biopsy‐anchored risk stratification using routinely available variables. AKRiS and RRS remain important because of their broader validation and performance across different populations and prediction horizons [6, 7].

Prognostic scores should complement, not replace, clinical assessment. Therapeutic decisions in AAV depend on disease severity, the pattern of organ involvement, comorbidities, and individual patient characteristics [28, 29]. In patients with severe or rapidly progressive kidney involvement, treatment decisions—including consideration of plasma exchange—should be guided by the overall clinical presentation and current guideline recommendations [30], rather than by the predicted risk from a prognostic score alone. In this context, ANCLA may provide additional information for early prognostic stratification while remaining distinct from treatment‐selection tools.

Performance also depends on prediction horizon and case mix. Our cohort’s advanced kidney dysfunction and frequent early ESKD supported evaluation at 1 and 2 years. Long‐term studies show that score performance can vary over follow‐up [10] and that ESKD burden accumulates over time, with kidney function at diagnosis and during follow‐up strongly associated with subsequent kidney failure [31]. Independent multicenter validation is therefore required to determine whether ANCLA’s short‐term performance is maintained over longer horizons.

## 5. Limitations

Limitations include the retrospective single‐center design and referral‐center case mix, which may limit generalizability; relatively short follow‐up, which restricts inference beyond 2 years; and evaluation of ANCLA in its derivation cohort, with potential derivation‐sample optimism [9]. Accordingly, these results represent comparative benchmarking rather than external validation. Formal calibration was limited to ANCLA because the comparator systems do not provide directly applicable individual absolute‐risk equations at these horizons. Longitudinal treatment response and relapse were not modeled, and treatment adjustment was avoided because of substantial potential for confounding by indication.

In summary, ANCLA showed the most favorable short‐term discrimination for ESKD in this high‐risk Latin American cohort, while AKRiS and RRS also performed strongly [6, 7]. Independent multicenter external validation should assess transportability and long‐term performance.

## Author Contributions

Research idea and study design: Joaquín Rodelo‐Ceballos and Mayra Estacio; data acquisition, curation, and validation: Mayra Estacio, Carlos Muñoz‐Vahos, and Alejandra Taborda; histopathologic review and validation: Alejandra Taborda; data analysis and interpretation: Joaquín Rodelo‐Ceballos, Mauricio Restrepo‐Escobar, and Alejandra Taborda; statistical analysis: Mauricio Restrepo‐Escobar; drafting the manuscript: Joaquín Rodelo‐Ceballos, Mauricio Restrepo‐Escobar, Mayra Estacio, Carlos Muñoz‐Vahos, and Alejandra Taborda; and critical revision of the manuscript for important intellectual content: Joaquín Rodelo‐Ceballos, Mauricio Restrepo‐Escobar, Mayra Estacio, Carlos Muñoz‐Vahos, and Alejandra Taborda.

## Funding

No funding was received for this research.

## Disclosure

All authors approved the manuscript. All authors agree to be accountable for all aspects of the work and to ensure that questions related to the accuracy or integrity of any part of the work are appropriately investigated and resolved.

## Ethics Statement

The study protocol was reviewed and approved by the Ethics Committee of Hospital San Vicente Fundación (HUSVF) under Act No. 24–2025 and by the Research Committee of Universidad de Antioquia. The study adhered to the provisions of Resolution No. 008430 of 1993 of the Colombian Ministry of Health and to the ethical principles of the 2013 Declaration of Helsinki. Given the retrospective nature of the study, based on the review of existing medical records and kidney biopsy reports, the requirement for informed consent was waived.

## Conflicts of Interest

The authors declare no conflicts of interest.

## Supporting Information

Additional supporting information can be found online in the Supporting Information section.

## Supporting information


**Supporting Information** Supporting Table S1. Baseline characteristics according to the composite outcome (ESKD or death). Supporting Table S2. Frequency of the composite outcome (ESKD or death) across prognostic model categories. Supporting Table S3. Paired AUC differences at 1‐ and 2‐year horizons. Supporting Table S4. Discrimination and calibration metrics at 1‐ and 2‐year horizons. Supporting Table S5. Summary of prognostic models and application rules.

## Data Availability

The data that support the findings of this study are available from the corresponding author upon reasonable request.
