# Supplementary material for: Comparative Performance of the ANCLA Risk Score and Established Prognostic Models for Predicting ESKD in ANCA‐Associated Glomerulonephritis: A Latin American Cohort
Source: Int J Nephrol. 2026 Oct 6;2026:5595479. doi: 10.1155/ijne/5595479 (PMC13640538; doi:10.1155/ijne/5595479)
Supplement: Supplementary file 1 [file IJNE-2026-5595479-s001.docx]

**Supplementary Material**

## Supplementary Table S1. Baseline characteristics according to the composite outcome (ESKD or death)

| **Variable** | **Total (n=164)** | **No composite (n=68)** | **Composite (n=96)** | **p value** |
| --- | --- | --- | --- | --- |
| Age, years, median (IQR) | 59.5 (42.5-68) | 56.5 (45.5-66) | 61 (40.5-69) | 0.493 |
| Female sex, n (%) | 77 (47.0) | 32 (47.1) | 45 (46.9) | 0.981 |
| BVAS, median (IQR) | 17 (14-20) | 16 (13-20) | 18 (15-21) | 0.093 |
| eGFR (CKD-EPI 2021), mL/min/1.73 m², median (IQR) | 15 (8-34) | 30 (15-50) | 11 (6-18) | <0.001 |
| Active urinary sediment, n (%) | 155 (94.5) | 64 (94.1) | 89 (94.7) | 0.877 |
| 24-hour proteinuria, mg/day, median (IQR) | 1749 (900-3265) | 1490 (871-2470) | 2165 (900-3808) | 0.061 |
| ANCA subtype, n (%) |  |  |  | 0.334 |
| MPO | 98 (60.0) | 42 (62.7) | 56 (57.7) |  |
| PR3 | 38 (23.2) | 17 (25.4) | 21 (21.6) |  |
| ANCA-negative | 28 (17.1) | 8 (11.9) | 20 (20.6) |  |
| Hemoglobin, g/dL, median (IQR) | 9 (7.9-10.4) | 10.0 (8.6-11.0) | 8.5 (7.5-9.7) | <0.001 |
| C-reactive protein, mg/dL, median (IQR) | 7.5 (2.8-13.8) | 8.4 (3.0-14.6) | 6.0 (2.0-13.0) | 0.365 |
| IV glucocorticoid pulses, n (%) | 156 (95.1) | 68 (100.0) | 88 (91.7) | 0.015 |
| Cyclophosphamide use, n (%) | 118 (72.0) | 55 (80.9) | 63 (65.6) | 0.032 |
| Rituximab use, n (%) | 21 (12.8) | 12 (17.7) | 9 (9.4) | 0.118 |
| Plasma exchange, n (%) | 40 (24.4) | 13 (19.1) | 27 (28.1) | 0.186 |
| Alveolar hemorrhage, n (%) | 34 (20.9) | 13 (19.1) | 21 (22.1) | 0.643 |
| Hypertension, n (%) | 84 (51.2) | 53 (52.5) | 31 (49.2) | 0.684 |
| Diabetes mellitus, n (%) | 26 (16.0) | 14 (14.0) | 12 (19.1) | 0.391 |
| Dyslipidemia, n (%) | 24 (14.7) | 15 (15.0) | 9 (14.3) | 0.900 |
| Heart failure, n (%) | 16 (9.8) | 12 (12.0) | 4 (6.4) | 0.238 |
| Cancer, n (%) | 4 (2.5) | 3 (3.0) | 1 (1.6) | 0.570 |
| Total glomeruli, median (IQR) | 18 (12-24) | 18 (12-24) | 18 (12-24) | 0.778 |
| Fibrous crescents, median (IQR) | 0 (0-1) | 0 (0-1) | 0 (0-1) | 0.894 |
| IFTA (global), %, median (IQR) | 20 (10-45) | 20 (10-30) | 38 (15-60) | <0.001 |
| Cellular crescents, median (IQR) | 2 (0-5) | 2 (1-5) | 2 (0-5) | 0.319 |
| Fibrocellular crescents, median (IQR) | 0 (0-1) | 0 (0-1) | 0 (0-2) | 0.277 |
| Fibrinoid necrosis, median (IQR) | 1 (0-2) | 1 (0-2) | 0 (0-2) | 0.042 |
| Globally sclerotic glomeruli, median (IQR) | 3 (1-9) | 2 (0-6) | 6 (2-11) | <0.001 |
| Glomerulosclerosis, %, median (IQR) | 22 (5-54) | 13 (0-35) | 44 (20-69) | <0.001 |
| Normal glomeruli, %, median (IQR) | 19 (2-40) | 28 (11-50) | 7 (0-17) | <0.001 |
| Arteritis, n (%) | 18 (11.2) | 16 (16.0) | 2 (3.3) | 0.013 |
| Atherosclerosis, n (%) | 75 (46.6) | 39 (39.0) | 36 (59.0) | 0.014 |

Baseline characteristics stratified by the composite outcome of ESKD or death during follow-up. Data are presented as median (IQR) or n (%). Statistical comparisons as in Table 1.

**Supplementary Table S2. Frequency of the composite outcome (ESKD or death) across prognostic model categories.**

| **Prognostic model** | **Risk category** | **Composite outcome (%)** |
| --- | --- | --- |
| Berden classification | Focal | 43.3 |
| Berden classification | Mixed | 56.0 |
| Berden classification | Crescentic | 56.1 |
| Berden classification | Sclerotic | 74.4 |
| Renal Risk Score (RRS) | Low risk | 29.6 |
| Renal Risk Score (RRS) | Intermediate risk | 52.2 |
| Renal Risk Score (RRS) | High risk | 75.4 |
| Mayo Clinic Chronicity Score (MCCS) | Minimal | 38.7 |
| Mayo Clinic Chronicity Score (MCCS) | Mild | 48.7 |
| Mayo Clinic Chronicity Score (MCCS) | Moderate | 62.8 |
| Mayo Clinic Chronicity Score (MCCS) | Severe | 76.3 |
| ANCLA score | Low risk | 26.8 |
| ANCLA score | Moderate risk | 46.3 |
| ANCLA score | High risk | 73.2 |
| ANCLA score | Very high risk | 87.5 |
| AKRiS | Low risk | 37.7 |
| AKRiS | Moderate risk | 62.2 |
| AKRiS | High risk | 72.0 |
| AKRiS | Very high risk | 95.8 |

Observed frequency of the composite outcome (ESKD or death) within each model-defined risk category.

**Supplementary Table S3. Paired AUC differences at 1- and 2-year horizons**

| **Horizon** | **Comparator** | **Delta AUC** | **Bootstrap 95% CI** | **DeLong p** | **Holm-adjusted p** |
| --- | --- | --- | --- | --- | --- |
| 1 year | AKRiS | +0.048 | -0.001 to +0.103 | 0.065 | 0.065 |
| 1 year | RRS | +0.062 | +0.011 to +0.115 | 0.019 | 0.039 |
| 1 year | MCCS | +0.150 | +0.063 to +0.241 | 0.001 | 0.003 |
| 1 year | Berden | +0.192 | +0.105 to +0.282 | <0.001 | <0.001 |
| 2 years | AKRiS | +0.053 | +0.002 to +0.108 | 0.048 | 0.050 |
| 2 years | RRS | +0.057 | +0.008 to +0.110 | 0.025 | 0.050 |
| 2 years | MCCS | +0.163 | +0.074 to +0.255 | <0.001 | 0.001 |
| 2 years | Berden | +0.203 | +0.112 to +0.298 | <0.001 | <0.001 |

Pairwise comparisons were performed using a common paired sample for ANCLA and the corresponding comparator at each horizon. ΔAUC was calculated as AUC_ANCLA − AUC_comparator; positive values favor ANCLA. Because the paired comparisons and the model-specific analyses may include different sets of patients, the ΔAUC values may not equal the direct subtraction of the AUCs reported in Supplementary Table S4. Patients censored before the corresponding horizon were excluded.

**Supplementary Table S4. Discrimination and calibration metrics at 1- and 2-year horizons**

| **Model** | **C-index** | **AUC 1y** | **AUC 2y** | **Cal intercept 1y** | **Cal slope 1y** | **Cal intercept 2y** | **Cal slope 2y** |
| --- | --- | --- | --- | --- | --- | --- | --- |
| ANCLA | 0.775 | 0.862 | 0.875 | 0.2 | 1.1 | 0.224 | 1.074 |
| AKRiS | 0.748 | 0.826 | 0.828 |  |  |  |  |
| RRS | 0.721 | 0.824 | 0.833 |  |  |  |  |
| MCCS | 0.66 | 0.729 | 0.72 |  |  |  |  |
| Berden | 0.64 | 0.705 | 0.726 |  |  |  |  |

Comparative discrimination of prognostic models for ESKD using Harrell's C-index (time-to-event framework) and horizon-specific AUC estimates at 1 and 2 years. Calibration intercept and slope estimates are reported only for ANCLA, given the availability of an underlying prognostic equation for absolute risk estimation.
Abbreviations: AUC, area under the receiver operating characteristic curve; C-index, concordance index; ESKD, end-stage kidney disease.

**Supplementary Table S5. Summary of prognostic models and application rules**

| **Model** | **Inputs required** | **Score computation** | **Risk categories used** | **Notes (implementation)** |
| --- | --- | --- | --- | --- |
| Berden histopathologic classification (2010) | Percentage of normal glomeruli, cellular/fibrocellular/fibrous crescents, globally sclerotic glomeruli | Class assignment based on glomerular lesion distribution: focal (≥50% normal), crescentic (≥50% crescents), sclerotic (≥50% globally sclerotic), mixed (remaining) | Focal; Mixed; Crescentic; Sclerotic | Applied using the original definitions and thresholds. Crescents include cellular, fibrocellular and fibrous as in original classification. |
| Mayo Clinic Chronicity Score (MCCS) / chronicity grading | Glomerulosclerosis, IFTA, arteriosclerosis (semiquantitative grading) | Sum of chronic injury components according to MCCS grading scheme (0–12 points) | Minimal (0–1); Mild (2–4); Moderate (5–7); Severe (≥8) | Chronicity components were graded from standardized pathology assessment. Categorization followed published cutoffs. |
| Renal Risk Score (RRS, 2018) | % normal glomeruli; IFTA (%); eGFR at biopsy | Points: normal glomeruli (>25%=0; 10–25%=4; <10%=6) + IFTA (≤25%=0; >25%=2) + eGFR (>15=0; ≤15=3). Total 0–11. | Low (0); Intermediate (2–7); High (8–11) | Computed exactly per original scoring rules using baseline values obtained near biopsy. |
| Improved Kidney Risk Score in ANCA-associated vasculitis (AKRiS, 2024) | Serum creatinine (µmol/L); % normal glomeruli; IFTA (% of cortex) | Points: creatinine (<250=0; 250–450=4; >450=11) + normal glomeruli (>25=0; 10–25=4; <10=7) + IFTA (<25=0; ≥25=3). Total 0–21. | Low (0–4); Moderate (5–11); High (12–18); Very high (21) | Applied exactly as described in the original publication. |
| ANCLA score (Latin American cohort) | Age; baseline eGFR; 24-hour proteinuria (g/day); % normal glomeruli | Risk (%) = 100 × exp[age × (−0.01386416) + eGFR × (−0.06736363) + 24-hour proteinuria (g/day) × 0.1840068 + normal glomeruli (%) × (−0.02291556)]. | Cohort-based quartiles: Low; Moderate; High; Very high | Analyzed continuously for discrimination. Cohort-based quartiles were used in Table 1, Table 2, and Kaplan-Meier analyses. |

Definition of variables, scoring rules, and risk categories used to implement each prognostic system according to the original publications. Abbreviations: eGFR, estimated glomerular filtration rate; IFTA, interstitial fibrosis/tubular atrophy.
